# How Global Is the Global Biodiversity Information Facility?

**DOI:** 10.1371/journal.pone.0001124

**Published:** 2007-11-07

**Authors:** Chris Yesson, Peter W. Brewer, Tim Sutton, Neil Caithness, Jaspreet S. Pahwa, Mikhaila Burgess, W. Alec Gray, Richard J. White, Andrew C. Jones, Frank A. Bisby, Alastair Culham

**Affiliations:** 1 School of Biological Sciences, Plant Science Laboratories, University of Reading, Whiteknights, Reading, United Kingdom; 2 School of Computer Science, Cardiff University, Cardiff, United Kingdom; University of Kansas, United States of America

## Abstract

There is a concerted global effort to digitize biodiversity occurrence data from herbarium and museum collections that together offer an unparalleled archive of life on Earth over the past few centuries. The Global Biodiversity Information Facility provides the largest single gateway to these data. Since 2004 it has provided a single point of access to specimen data from databases of biological surveys and collections. Biologists now have rapid access to more than 120 million observations, for use in many biological analyses. We investigate the quality and coverage of data digitally available, from the perspective of a biologist seeking distribution data for spatial analysis on a global scale. We present an example of automatic verification of geographic data using distributions from the International Legume Database and Information Service to test empirically, issues of geographic coverage and accuracy. There are over 1/2 million records covering 31% of all Legume species, and 84% of these records pass geographic validation. These data are not yet a global biodiversity resource for all species, or all countries. A user will encounter many biases and gaps in these data which should be understood before data are used or analyzed. The data are notably deficient in many of the world's biodiversity hotspots. The deficiencies in data coverage can be resolved by an increased application of resources to digitize and publish data throughout these most diverse regions. But in the push to provide ever more data online, we should not forget that consistent data quality is of paramount importance if the data are to be useful in capturing a meaningful picture of life on Earth.

## Introduction

The availability of biodiversity data is a major issue at a time of global habitat loss [1]. The largest single data portal is the Global Biodiversity Information Facility (GBIF). GBIF is an intergovernmental organisation providing “an internet accessible, interoperable network of biodiversity databases and information technology tools”[2], with a “mission to make the world's biodiversity data freely and universally available via the Internet” [3] and has been described as a “cornerstone resource” [4]. Currently, the GBIF portal provides access to biodiversity information from museums, herbaria and other organisations around the globe. There are 199 host institutions providing more than 120 million records (http://www.gbif.org/ accessed 6^th^ March 2007). While the database as a whole is large, its coverage is patchy, with some areas and taxa well covered while others are absent. Here we present an exemplar assessment of these data using the third largest flowering plant family, the Leguminosae, to evaluate both the coverage and accuracy of electronically recoverable point distribution data.

One of GBIF's strategic objectives is to “enable scientific research that has never before been possible” [3]. These data are an important source of information for the biological researcher. The data can be used for, amongst other things; taxonomic revisions [5], environmental niche modelling [6], compiling redlists of threatened species [7] and biodiversity assessment [8]. See Graham *et al.* [9] and Suarez and Tsutsui [10] for more detailed reviews of additional uses of museum specimen data. This work facilitates biodiversity policy- and decision-making [3].

The patchy coverage of GBIF data, even over small geographic scales was illustrated by a small scale environmental niche modelling study of *Cyclamen* [11] that compared data from GBIF with detailed extent of occurrence maps to predict lineage extinction risk. The poor quality of some data provided by GBIF was highlighted by a study of the effects of palaeohistoric climate change on the evolution and current distribution of *Drosera* [12]. This study used a global species database to filter geographic records to address this issue.

The value of GBIF data points lies in the uses that can be made of sets of such points on a comparative basis, as in taxonomic and biogeographic analyses. Here we are exploring:

1. Geographic accuracy: whether details of specimen location are given with consistent accuracy;
2. Geographic sampling consistency: whether specimens are recorded without regional bias.

For all data attached to a record, the reliance on a correct name is absolute. An incorrect name is positively misleading because it may link real data to the wrong taxon. Names can be incorrect due to misidentification or the application of a name that is not accepted under the taxonomy used by the researcher.

One of the most important pieces of information held for a specimen is the field-collection locality. This permits mapping, as well as studies of distribution, biogeography and conservation [9]. The potential benefit of these distribution data is well known, as are the problems. There are many articles outlining the theoretical errors associated with distribution data from museum collections [9], [13], [14], but few which test these errors on a large scale with real data.

We have explored the global point data provided by GBIF using the International Legume Database & Information Service (ILDIS) to validate point data, both taxonomically and spatially.

This permits us to answer:

- Are these data geographically plausible?
- What are the geographical biases inherent in these data?
- To what extent is it practical or possible to validate these data nomenclaturally?

ILDIS is a global species database providing expert taxonomic and area occurrence data for the twenty thousand species of Leguminosae [15], one of the largest families of flowering plants, often considered as representative of global plant biodiversity [16].

## Materials and Methods

### Data gathering–Georeferenced data

The GBIF portal was queried for georeferenced data (i.e. those with latitude/longitude coordinates) using custom web-scraping scripts in a batch process. These queries used all species names from ILDIS version 9.0, including synonyms but excluding the very few names with pro-parte synonyms, or marked ‘invalid’. This consisted of 31,086 ‘valid’ names representing 20,003 species. (Data accessed 26–28/08/2005).

### Data gathering-Georectifying non georeferenced records with Biogeomancer

Many GBIF records lack coordinate data. To discover how many of these might be useable, if georectified, we tested five species with wide distributions (*Inga edulis*, *Acacia farnesiana*, *Adenocarpus complicatus*, *Crotalaria goreensis* and *Mimosa pigra*). Georectification used Biogeomancer Classic's batch submission process (http://www.biogeomancer.org/) for deduction of latitude/longitude coordinates from place names.

### Name validation

Only records with an exact match on Genus+species+Author were analysed. ILDIS synonymy was used to attach the accepted species name to each record. This effectively combined data attached to different synonyms into a single dataset for the currently accepted taxon. It is noted that GBIF use a name validation process based on Species2000 & ITIS Catalogue of Life for which ILDIS provides the Legume names.

### Spatial validation

We analysed only georeferenced records. All regional analysis used the Taxonomic Database Working Group Geography Standard version 2.0 level 4 areas (TDWG4), ([17] data available as vector maps at http://www.rbgkew.org.uk/gis/tdwg). This is essentially a country-level classification, with large countries and island groups sub-divided. Records were treated as ‘valid’ if the georeferenced point fell within a TDWG4 area in which ILDIS records species-level occurrence. Spatial analysis and data manipulation to perform this validation used a PostgreSQL database (http://www.postgresql.org) with the Postgis plugin (http://postgis.refractions.net/). Maps were generated using the Quantum GIS mapping software (http://qgis.org). Chapman [18] discussed a broad range of techniques to validate spatial data, including this approach. Yesson & Culham [12] have used this approach to filter GBIF data for use in environmental niche modelling.

## Results

**Figure 1:**
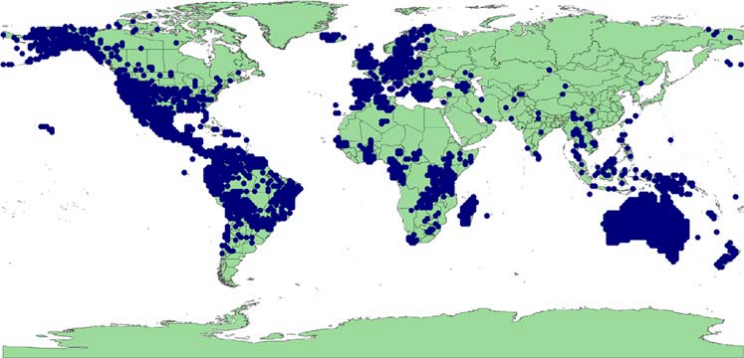
All valid points collected from GBIF database

The search of GBIF returned 630,871 records with georeferenced data for Legumes (appendix S1 contains the list of source institutions). At least one georeferenced record was found for 6,147 species representing 31% of all Legume species recognised by ILDIS. 533,026 records (84%) were geographically validated by ILDIS distribution data (Figure 1), accounting for 5,423 species (27% of Legumes). Therefore 724 species (3.6%) consist only of records that failed validation.

### Exclusions

**Figure 2:**
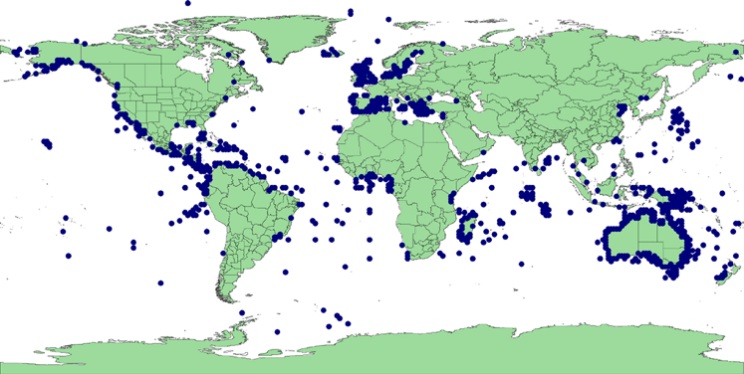
GBIF points classified ‘In the sea’

**Figure 3:**
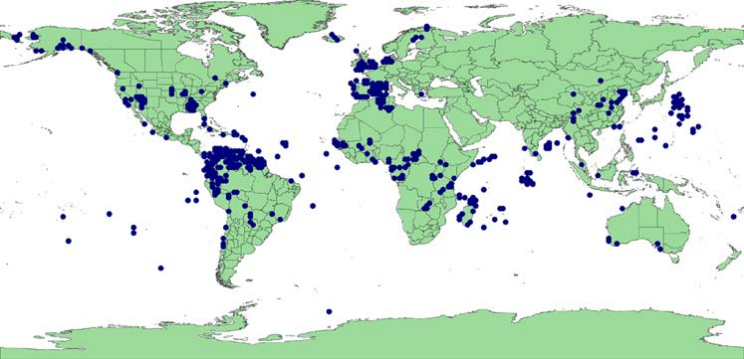
GBIF points classified ‘Lat/Long error’

**Figure 4:**
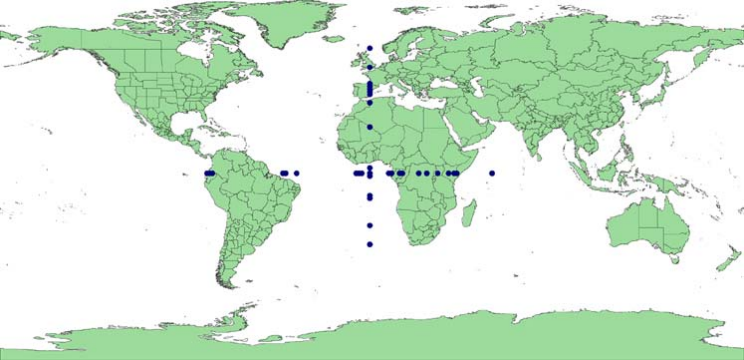
GBIF points classified ‘Lat/Long exactly zero’

97,845 records (16%) were classed as geographically invalid. On inspection, there appeared to be several reasons for the invalid classification, which were given the following categories:

- ‘In the sea’: coordinates that did not project onto land (there are no marine legumes) (82% of invalid records) (Figure 2). The vast majority of these occur along coastlines and may represent uncertainty due to insufficient resolution in the recording of co-ordinates.
- ‘Lat/Long error’: reversing the sign of one or both the latitude or longitude values or swapping the latitude and longitude values produced a valid locality (40%). Figure 3 reveals an inverted silhouette of Morocco over Algeria reflecting an error in processing the sign of the longitude of records sourced from the University of Reading. There are also a large number of likely Australian records off the east coast of Japan due to an incorrect sign for the latitude of these records. However, this set also includes many records close to the equator & meridian that are, in reality, near valid resolution uncertainties that become falsely validated by reversal of the sign. For example, records from the east coast of the UK are validated by sign reversal which puts the points well inland.
- ‘Lat/Long zero’: latitude or longitude is exactly zero, suggesting missing data misinterpreted as real data (1%). Note that real points can occur both on the equator and the prime meridian so that some rejected points could be genuine (Figure 4).

**Figure 5:**
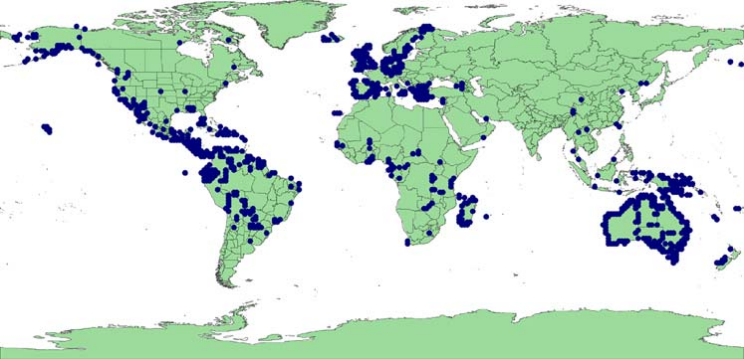
GBIF points classified ‘Near valid’

**Figure 6:**
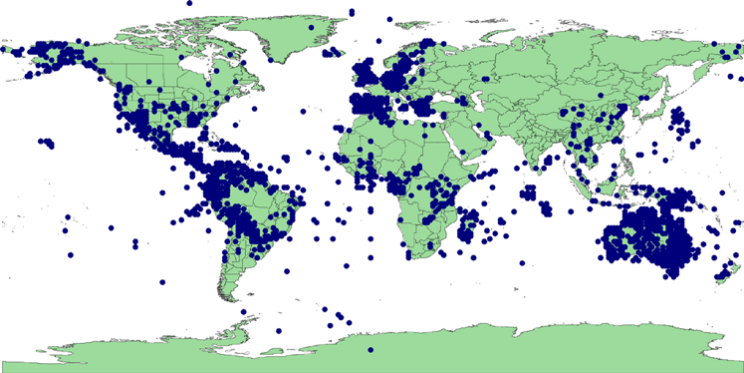
GBIF points classified ‘Far from valid’

These categories are not mutually exclusive, but can be simplified into two classes which are mutually exclusive:

- ‘Near Valid’: the observed point is within 0.5 degrees of a valid area (83%) (Figure 5). This includes many of the ‘in the sea’ category, or points close to the border of valid areas, and may be caused by limited resolution in the recording of co-ordinates. The choice of 0.5 degrees is arbitrary, using 0.1 degree reduces this proportion to 71%, and if we increase resolution to 1 minute then only 23% of records are ‘near valid’.
- ‘Far from valid’: the observed point is beyond 0.5 degrees of a valid area (17%) (Figure 6). These are the most worrying incorrect records, and include many of the genuine lat/long errors. The use of 0.1 degree increases the proportion to 29% and 1 minute gives 77%.

### Biogeomancer

The five exemplar species used to evaluate Biogeomancer had 2,881 GBIF records, of which 43% were already georeferenced. 355 (12%) of these were successfully georectified. Only 112 (4%) of these were ‘new’ coordinates for records not georeferenced in GBIF. The georectified coordinates were identical to those provided by GBIF in only 3 cases, but 76% were within 0.5 degrees. 94% of the georectified data passed ILDIS validation. Based on these five examples, we extrapolate 59,000 additional records could have been georeferenced and added to our analysis. Given that this would increase our data set by less than 10%, the considerable time input in processing these records was not justified in this instance.

### Data providers

**Figure 7:**
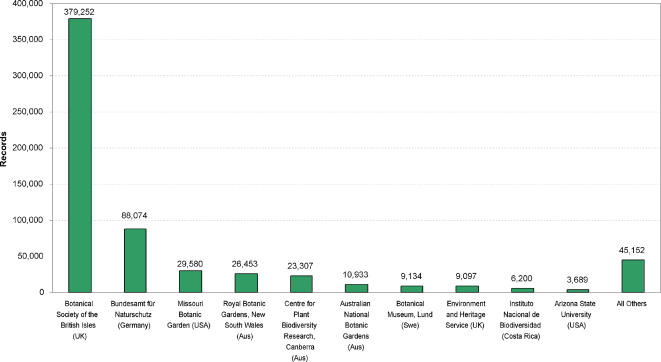
The top 10 data suppliers of Legume records

**Figure 8:**
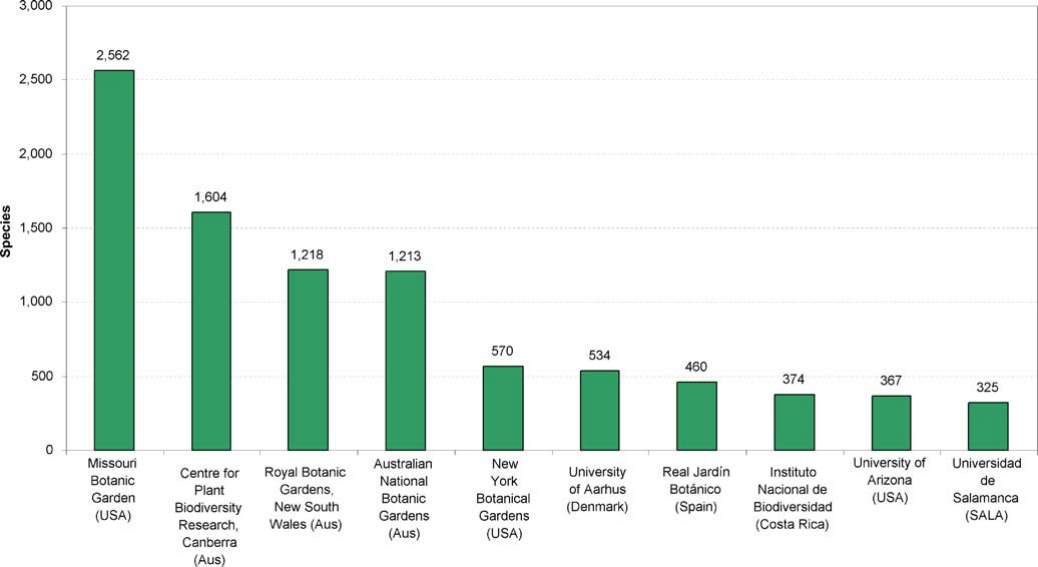
The top 10 data suppliers of Legume species

Nearly 60% of records we recovered come from the UK National Biodiversity Network (NBN) (Table 1 & Figure 7). The second largest data source, Bundesamt für Naturschutz, provided a further 16%. These two suppliers provide gridded presence data for species, based on surveys rather than label information directly linked to herbarium/museum specimens. These two sources only provide data for 137 species. In contrast Missouri Botanical Garden provides 4% of records but includes 2,562 species (Figure 8).

**Table 1:** Top GBIF data providers for Legume data. Note: the species count is not cumulative as species data can be from more than one provider.

Country-Provider	verified records (rank)	% total	% records verified	valid species	% total species
UK-National Biodiversity Network	314,959	59.1%	83.0%	110	2.0%
Germany-Bundesamt für Naturschutz	83,943	15.7%	95.3%	73	1.3%
Australia-National Herbarium of New South Wales	24,950	4.7%	94.3%	1,140	21.0%
Australia-Centre for Plant Biodiversity Research	20,361	3.8%	87.4%	1,604	29.6%
USA-Missouri Botanic Gardens	20,174	3.8%	68.2%	2,562	47.2%
Australia-National Botanic Garden	10,075	1.9%	92.2%	1,213	22.4%
Sweden-Lund Botanical Museum	6,845	1.3%	74.9%	278	5.1%
UK-Environment and Heritage Service	4,868	0.9%	53.5%	25	0.5%
USA-Arizona State University	3,479	0.7%	94.3%	178	3.3%
Costa Rica-Instituto Nacional de Biodiversidad	3,176	0.6%	77.8%	170	3.1%
Sweden-GBIF-SE:ArtDatabanken	3,150	0.6%	87.3%	60	1.1%
All Others	37,046	7.0%	92.4%	-	-
**Total**	**533,026**	**100%**	**92.4%**	**5,423**	**100%**

### Geographic range

**Figure 9:**
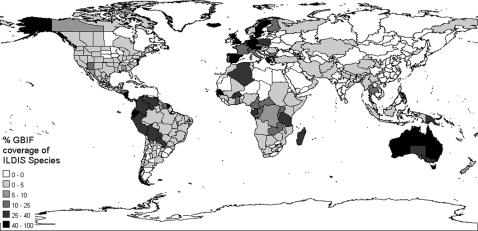
Global Legume coverage from GBIF data per TDWG level 4 area.

**Figure 10:**
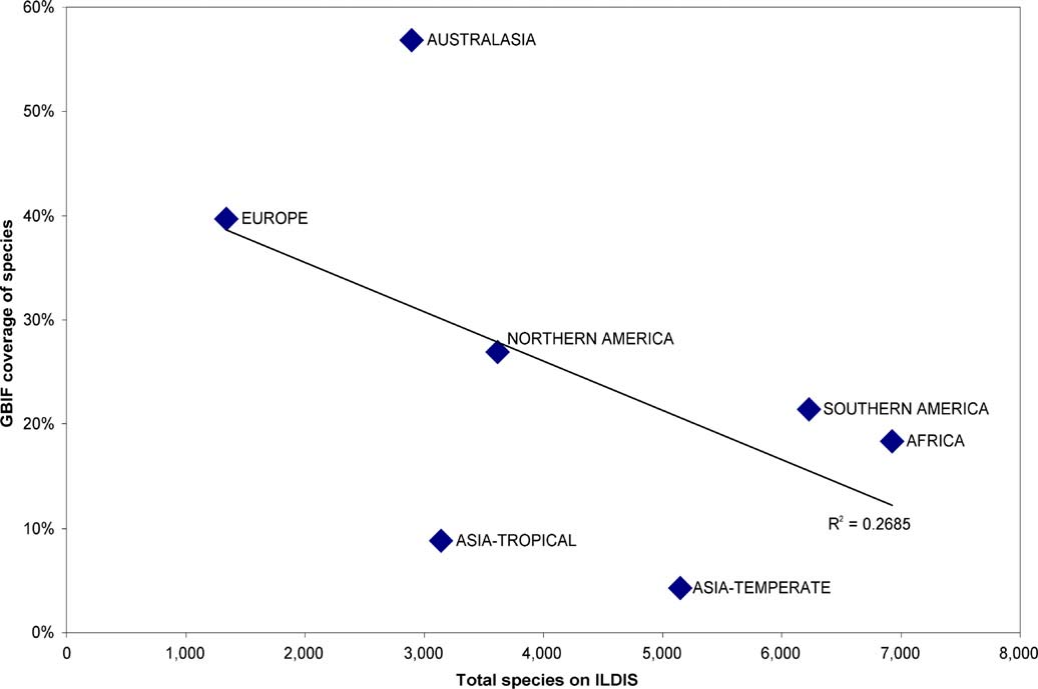
Species coverage on GBIF at a continental scale (TDWG level 1 continents).

The ILDIS database provides the number of species present in each TDWG4 area, therefore counting GBIF species in each area permits quantification of species coverage by area. The global species coverage is 27%, but the average coverage per area is just 4%, the standard deviation is 11% demonstrating how much this varies by area (Figure 9 & Table 2). Western Europe, Australia and Central America have good coverage. Over 500 areas have<5% coverage. Some areas with poor coverage are considered globally important for biodiversity [19]. For example the winter-rainfall diversity hotspot of the Cape floristic region has data for only 35 records, which compares poorly with the 7,499 records for the winter-rainfall diversity hotspot of Southwest Australia (Table 3). On a continental scale there is a negative correlation of species coverage with species diversity (Figure 10).

**Table 2:** Species coverage of GBIF records using ILDIS to define total species numbers

TDWG Level 4 Name	Country	GBIF Records	GBIF Species	ILDIS Species	% Coverage
Sweden	Sweden	5,897	69	100	69%
East Aegean Is.	Greece	1,403	140	224	63%
Great Britain	United Kingdom	319,915	110	183	60%
Spain	Spain	12,857	366	623	59%
Costa Rica	Costa Rica	6,013	270	479	56%
Greece	Greece	5,031	259	491	53%
Ecuador	Ecuador	3,339	201	383	52%
Nicaragua	Nicaragua	2,545	136	262	52%
New South Wales	Australia	20,776	493	964	51%
Western Australia	Australia	11,855	873	1734	50%
Cocos I.	Costa Rica	1	1	2	50%
Germany	Germany	82,831	87	182	48%
Kriti	Greece	454	112	237	47%
Queensland	Australia	8,062	536	1170	46%
French Guyana	French Guyana	2,401	188	417	45%
Alaska	United States	1,126	27	62	44%
Iceland	Iceland	76	8	19	42%
Tasmania	Australia	451	50	120	42%
Senegal	Senegal	440	123	298	41%
Northern Territory	Australia	6,244	342	848	40%
**Total (609 areas)**		**533,026**	**5,423**	**20014**	**27%**

**Table 3:** GBIF data for global the hotspots of Mittermeier *et al*. [19]

Hotspot Name*	GBIF records (rank)	GBIF Species	% Total	% Total
Mediterranean Basin	18,156	515	3.4%	9.5%
Mesoamerica	9,714	595	1.8%	11.0%
Southwest Australia	7,499	598	1.4%	11.0%
Tropical Andes	2,918	472	0.5%	8.7%
Madrean Pine-Oak Woodlands	1,413	246	0.3%	4.5%
Tumbes-Choco-Magdalena	1,259	224	0.2%	4.1%
New Zealand	1,023	22	0.2%	0.4%
Madagascar and the Indian Ocean Islands	762	217	0.1%	4.0%
Eastern Afromontane	567	265	0.1%	4.9%
Atlantic Forest	487	150	0.1%	2.8%
Guinean Forests of West Africa	303	136	0.1%	2.5%
Cerrado	240	117	0.0%	2.2%
Coastal Forests of Eastern Africa	222	118	0.0%	2.2%
Indo-Burma	157	106	0.0%	2.0%
California Floristic Province	115	67	0.0%	1.2%
East Melanesian Islands	104	37	0.0%	0.7%
Polynesia-Micronesia	87	25	0.0%	0.5%
Sundaland	69	40	0.0%	0.7%
Caribbean Islands	45	35	0.0%	0.6%
Caucasus	44	37	0.0%	0.7%
Cape Floristic Region	32	25	0.0%	0.5%
Irano-Anatolian	32	24	0.0%	0.4%
Wallacea	32	12	0.0%	0.2%
Horn of Africa	29	14	0.0%	0.3%
Chilean Winter Rainfall and Valdivian Forests	22	13	0.0%	0.2%
Philippines	15	14	0.0%	0.3%
Mountains of Southwest China	11	8	0.0%	0.1%
New Caledonia	11	4	0.0%	0.1%
Japan	5	1	0.0%	0.0%
Mountains of Central Asia	5	4	0.0%	0.1%
Succulent Karoo	3	2	0.0%	0.0%
Western Ghats and Sri Lanka	3	3	0.0%	0.1%
Himalaya	1	1	0.0%	0.0%
Maputaland-Pondoland-Albany	1	1	0.0%	0.0%
**Total Inside a Hotspot**	**45,386**	**3,199**	**8.5%**	**59.0%**
**Outside Hotspots**	**487,640**	**3,644**	**91.5%**	**67.2%**
**Total**	**533,026**	**5,423**	**100.0%**	**100.0%**

### Species completeness

Many species are found in more than one area. Of our 5,423 species with some GBIF data, some 68% are missing observations from one or more areas they are known to inhabit. Of those with species complete TDWG area coverage on GBIF, some 79% are endemic to a single area. This demonstrates that a crude measure such as number of species included in GBIF will still give a misleadingly optimistic impression of comprehensive data coverage.

The results section should provide details of all of the experiments that are required to support the conclusions of the paper. There is no specific word limit for this section. The section may be divided into subsections, each with a concise subheading. Large datasets, including raw data, should be submitted as supporting information files; these are published online alongside the accepted article. We advise that the results section be written in past tense.

## Discussion

GBIF provides more than half a million Legume records covering 31% of species, all freely available to a global audience. The majority of these data appear to be geographically accurate. An important part of the GBIF mission is the repatriation of data for specimens from developing countries [2]. In our study the vast majority (86%) of data for Legumes both come from, and apply to, three developed countries (UK, Germany, Australia). Almost 60% of these data come from one supplier, in a single country, the UK, which is not noted for its Legume diversity.

Using ILDIS ensured that only verified names were used in record selection, but confirmation of the identity of individual specimens cannot be automated at present. Correct specimen identification is ultimately the responsibility of the source data provider. If a misidentification creates an observation outside the known distribution then the validation procedure used in this study will exclude it. Furthermore, if our distribution data is incomplete, then real observations could be discarded. The Russian elements of the ILDIS geography are particularly incomplete (Yuri Roskov pers. comm.). In addition, the ILDIS database does not yet provide a complete synonymy for every species; this may have restricted the total record count.

**Figure 11:**
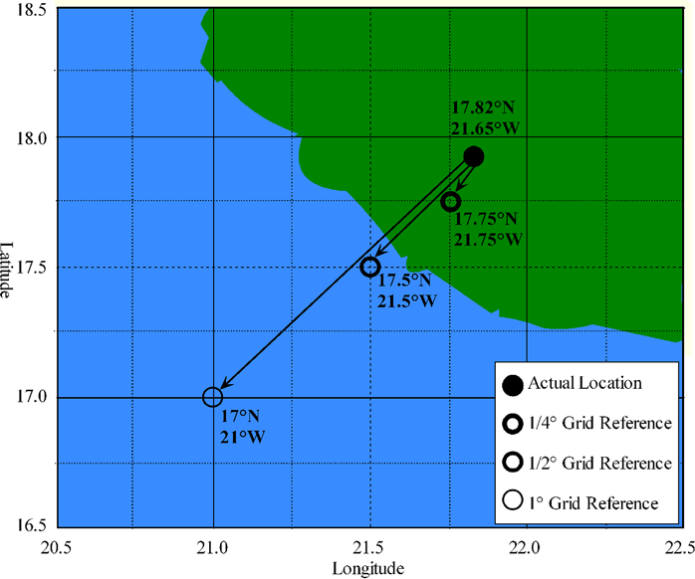
Hypothetical example of a point near a coastline which cross the land/sea barrier when referenced on grids of ¼, ½, and 1 degree resolution. Co-ordinates are displayed by points.

The majority (83%) of excluded records were geographically near to known valid areas and are likely to be good observations with poor geographic resolution. These are typically coastal observations where scale of resolution (Figure 11) causes the point to be projected into the sea. This type of scaling problem could equally occur entirely within a valid TDWG4 area, this is undetectable using our validation system. However, it may be possible to use these ‘near valid’ coastal records in an analysis by assuming the nearest land to be the ‘real’ location. Our conservative approach could have led to up to 13% of valid points being excluded.

The survey data from the UK are presence/absence mapping on a 10 km^2^ grid. Grid-based data are likely to have well-defined error limits (set by the bounds of the grid square). Herbarium data are usually based on individual Lat./Long. records either from GPS or via georeferencing of localities after collection. Effectively these data are also on a grid, governed by the resolution of the Lat./Long. data but there is a greater variability in grid size. This variability can potentially range from very precise (e.g. degrees, minutes and seconds) to imprecise (e.g. just degrees). Referencing a grid square of any size by a point within the grid (i.e. centre, or lower left corner) can result in the grid reference falling outside a valid area, while at least one part can overlap a valid area (Figure 11). Therefore an accurate grid reference can be invalidated purely due to issues of grid square size. This problem can be confounded by limits to the resolution of the underlying map.

Analysing Legumes permits geographic validation via ILDIS. Where such geographic validation is not available the majority of inaccurate records can be filtered by exclusion of points that do not project onto land, (at least for land-bound species).

Many analytic techniques require multiple observations from a representative sample to provide robust results. Many Legume species with georeferenced data have just a single observation (1,205 species). The 724 species excluded because none of their points passed validation largely fall in this category. Only 2,098 species are represented by 10 or more records.

GBIF data for Legumes are a geographically biased sample. Large parts of the globe, including hotspots of biodiversity in Africa and Asia, are data deficient. These gaps could most readily be filled by making existing databases widely available, and by digitisation of the major herbarium collections.

At present relatively few herbaria provide data to GBIF. Of the 11 large herbaria listed in Table 4, five had no specimens available via the GBIF portal and only 2 had more than 10% of their total specimens accessible. Only 6% of the 61,000,000 specimens from these institutions are accessible. In contrast, much new collecting is based in smaller herbaria. Databasing these collections, upon accession, could be a more valuable exercise for monitoring current frequency and distribution of species, due to the usually higher data quality associated with new collections.

**Table 4:** Large herbaria and their contribution to GBIF at the time of this analysis.

Code	Name of Herbarium	Country	Specimens *	GBIF totals #	% Total
P	Muséum National d'Histoire Naturelle, Paris	France	7,500,000	448,437	6%
K	Royal Botanic Gardens, Kew	UK	7,000,000	0	0%
NY	New York Botanical Garden	USA	7,000,000	91,037	1%
G	Conservatoire et Jardin botaniques de la Ville de Genève	Switzerland	6,000,000	202,855	3%
LE	V. L. Komarov Botanical Institute, St. Petersburg	Russia	5,770,000	0	0%
MO	Missouri Botanical Garden	USA	5,522,000	1,966,000	36%
BM	Natural History Museum, London	UK	5,200,000	232,418	4%
GH	Harvard University, Massachusetts	USA	5,005,000	0	0%
S	Swedish Museum of Natural History, Stockholm	Sweden	4,400,000	617,047	14%
US	Smithsonian Institution, Washington DC	USA	4,340,000	0	0%
MPU	Université Montpellier	France	4,000,000	0	0%
**Total**			**61,737,000**	**3,557,794**	**6%**

* Source: Index Herbariorum http://sciweb.nybg.org/science2/IndexHerbariorum.asp Accessed 10/2005. # Source: http://www.gbif.org Accessed 10/2005. Note: K now has c. 140,000 records, GH has c.220,000 records, and US has c.766,000 records on GBIF (09/2007), some other institutions have increased their online records substantially during the past 24 months.

This snapshot of data available via GBIF illustrates the generic patterns in the data, including accuracy and coverage. Coverage is improving gradually as more data providers come on line. The issue of synonymy is being addressed under the new, improved GBIF data portal.

### Conclusion

The GBIF point data are largely correct: 84% passed our conservative criteria. A serious problem is the uneven coverage of both species and areas in these data. It is possible to retrieve large numbers of accurate data points, but without appropriate adjustment these will give a misleading view of biodiversity patterns. Coverage associates negatively with species richness. There is a need to focus on databasing mega-diverse countries and biodiversity hotspots if we are to gain a balanced picture of global biodiversity. A major challenge for GBIF in the immediate future is a political one: to negotiate access to the several substantial biodiversity databases that are not yet publicly and freely available to the global science community. GBIF has taken substantial steps to achieve its goals for primary data provision, but support is needed to encourage more data providers to digitise and supply their records.

## Supporting Information

Appendix S1 List of institutions providing specimen data used in this analysis
(0.03 MB XLS)
